# Treatment with Lanreotide Depot Following Octreotide Long-Acting Release Among Patients with Gastroenteropancreatic Neuroendocrine Tumors

**DOI:** 10.1089/pancan.2018.0013

**Published:** 2018-10-01

**Authors:** Muhammad Wasif Saif, Julie Fu, Melissa H. Smith, Barbara Weinstein, Valerie Relias, Kevin P. Daly

**Affiliations:** ^1^ Department of Medical Oncology, Tufts Cancer Center–Tufts Medical Center, Boston, Massachusetts.; ^2^ Department of Pathology, Tufts Cancer Center–Tufts Medical Center, Boston, Massachusetts.; ^3^ Department of Invasive Radiology, Tufts Cancer Center–Tufts Medical Center, Boston, Massachusetts.

**Keywords:** chromogranin, gastroenteropancreatic neuroendocrine tumors, lanreotide depot, octreotide long-acting release

## Abstract

**Objective:** To examine patients with metastatic gastroenteropancreatic neuroendocrine tumors (GEP-NETs) who receive sequential treatment with somatostatin analogs.

**Materials and Methods:** This retrospective chart review examined lanreotide depot/autogel tolerability and efficacy among GEP-NET patients who received lanreotide after octreotide long-acting release (LAR) at Tufts University Medical Center. Information obtained included background patient characteristics, dosing, adverse events (AEs), radiologic response, and biochemical markers.

**Results:** Patients (*n* = 16; 43–81 years; mean age, 64.25 years; 11 female) with nonfunctional, low-grade GEP-NETs receiving octreotide LAR 30–60 mg were transitioned to lanreotide because of patient decision (*n* = 6), disease progression (*n* = 6), AEs (*n* = 2), poor tolerance (*n* = 1), and injection discomfort/pain (*n* = 1). Lanreotide doses started at 120 mg (*n* = 13), 90 mg (*n* = 1), or 60 mg (*n* = 2); 8 patients received concomitant therapies, mostly liver-directed (radiofrequency ablation/radioembolization). AEs associated with lanreotide experienced by ≥2 patients were fatigue, diarrhea, nausea, hypertension, pancreatic enzyme deficiency, and hyperglycemia. Radiologic treatment responses of the combination of lanreotide with other therapeutic modalities included complete response (*n* = 1), partial response (*n* = 5), and stable disease (*n* = 9). One patient had radiologic progression. Serum serotonin and chromogranin levels decreased, but urinary 5-hydroxyindoleacetic acid levels appeared relatively unchanged.

**Conclusion:** Among post-octreotide GEP-NET patients, including those with disease progression or poor octreotide tolerance, lanreotide alone or with concomitant therapies was well tolerated and associated with radiologic responses.

## Introduction

The incidence of neuroendocrine tumors (NETs) has increased markedly in the United States over the past several decades. The U.S. Surveillance, Epidemiology, and End Results (SEER) database, for example, shows a 6.4-fold increase in NETs from 1973 to 2012 (1.09–6.98 per 100,000 persons).^1^ For gastroenteropancreatic neuroendocrine tumors (GEP-NETs), the overall incidence was 3.56 per 100,000 persons between 2000 and 2012. Across all NET patients, median overall survival was the highest for NETs in the appendix (>30 years) and rectum (24.6 years), while NETs in the pancreas showed the lowest median survival (3.6 years).^1^

Evaluation of somatostatin receptor expression in human tissues is complicated by the fact that there are five receptor subtypes (SSTR1–SSTR5).^2^ Only a limited number of studies have comprehensively investigated the expression of all five of the SSTR subtypes, and thus knowledge regarding the correlation of their expression with clinical outcomes in NET is incomplete.^3^

An assessment of SSTR receptor subtypes in NETs revealed that 51% of cases highly expressed SSTR2, 47% SSTR1, 43% SSTR5, 36% SSTR4, and 23% SSTR3.^3^ A high expression of both SSTR1 and SSTR2 was found more frequently in pancreatic NETs and small intestinal NETs than in other NETs.^3^ It is important to note that octreotide and lanreotide, both of which are synthetic somatostatin analogs (SSAs), primarily bind to SSTR2 and SSTR5.^3^

The randomized, double-blind, placebo-controlled Controlled Study of Lanreotide Antiproliferative Response in Neuroendocrine Tumors (CLARINET) was the largest trial and one of the few trials conducted to compare long-acting lanreotide depot/autogel (*n* = 101, hereafter referred to as lanreotide) with placebo (*n* = 103) among patients with primary NETs in the pancreas, midgut, or hindgut.^4^ Progression-free survival (PFS) was the primary efficacy end-point of CLARINET. There was significantly prolonged PFS among patients treated with lanreotide (median PFS not reached vs. 18.0 months; *p* < 0.001; hazard ratio for progression/death for lanreotide vs. placebo, 0.47; 95% confidence interval [CI], 0.30–0.73).^4^ The estimated rates of PFS at 24 months were 65.1% (95% CI, 54.0–74.1) versus 33.0% (95% CI, 23.0–43.3) for the lanreotide and placebo groups, respectively. A greater number of patients in the placebo group (*n* = 58) than the lanreotide group (*n* = 30) had centrally assessed disease progression events. Two patients from each of the groups died. The most commonly reported treatment-related adverse events (AEs; >10% of patients) were diarrhea (26% in lanreotide group and 9% in placebo group) and abdominal pain (14% in lanreotide group and 2% in placebo group). A follow-up of CLARINET published 2 years later further demonstrated the long-term safety and tolerability of lanreotide.^5^

The treatment goals of NETs include suppressing tumor growth and controlling the symptoms of carcinoid syndrome.^6^ The primary objective of the Evaluating Lanreotide Efficacy and Safety as a Carcinoid-syndrome Treatment (ELECT) study was to determine whether there was a clinically meaningful difference between lanreotide (*n* = 59) and placebo (*n* = 56) groups in the use of daily short-acting subcutaneous rescue octreotide as a correlate of improved symptom control.^6^ Inclusion criteria were carcinoid (neuroendocrine) tumor or one on an unknown location with liver metastasis and a history of carcinoid syndrome. The adjusted mean (95% CI) percentage of days of rescue octreotide use during the 16-week double-blind phase was significantly lower in the lanreotide group (33.7% [25.0–42.4]) than the placebo group (48.5% [39.6–57.4]), with a between-group absolute difference of −14.8% (95% CI, −26.8 to −2.8; *p* = 0.017). From baseline to week 12, global health status/quality of life, gastrointestinal symptoms, and endocrine symptoms all improved among lanreotide-treated patients, while the placebo group experienced lesser improvements or no changes (95% CIs for adjusted treatment differences were wide and favored the lanreotide group). Excluding diarrhea and flushing, which were assessed separately in this study, no AE occurred in >9% of patients, and few AEs were serious (*n* = 2 [3.4%] for lanreotide vs. *n* = 5 [8.8%] for placebo).^6^ These results indicate that treatment with lanreotide could reduce the number of days that treatment with rescue octreotide is required.

Despite the fact that primary treatment for metastatic NETs often includes SSAs,^7,8^ the tolerability of sequential use of SSAs for GEP-NETs, including octreotide followed by lanreotide, has not been studied in depth. Although octreotide is not approved by the U.S. Food and Drug Administration (FDA) for treatment of GEP-NETs,^9^ a few studies have been conducted to measure the safety and efficacy of treatment for NETs. One of these was a randomized, placebo-controlled study that included patients with metastatic midgut NETs. The results showed that octreotide long-acting release (LAR; *n* = 42) stabilized tumor growth, prolonged time to tumor progression, and improved long-term survival compared with placebo (*n* = 43).^10^

Lanreotide 120 mg administered as a deep subcutaneous injection is approved by the FDA for treatment of GEP-NETs in patients with unresectable, well- or moderately-differentiated, locally-advanced, or metastatic GEP-NETs to improve PFS.^11^ Recently (September 2017), the same dosage was approved by the FDA for the treatment of carcinoid syndrome in adults to reduce the frequency of short-acting SSA rescue therapy.^11^ The objectives of this case series included assessing the safety, tolerability, and efficacy of lanreotide in patients with various GEP-NETs who were previously treated with octreotide LAR.

## Materials and Methods

### Study design

Institutional Review Board (IRB) approval was granted before the initiation of this retrospective chart review that was conducted at the Tufts University Medical Center. The procedures followed in this study were in accordance with the ethical standards of the responsible committee on human experimentation (institutional and national) and with the Declaration of Helsinki. Included patients had NETs and received lanreotide following octreotide LAR.

Each patient was evaluated by an oncologist or their nurse every 4 weeks before receiving subsequent lanreotide injections. All patients received deep subcutaneous injections of lanreotide, with the exception of one individual who received one injection of lanreotide by intramuscular route.

### Assessments and outcome measures

The information obtained from patient charts included demographic data, tumor stage/grade, SSA treatment/dose, AEs, radiologic response, and baseline/current levels of biochemical markers (chromogranin A [CgA], urinary 5-hydroxyindoleacetic acid [5-HIAA; primary metabolite of serotonin], serotonin, gastrin, pancreatic polypeptide, and adrenocorticotropic hormone). Treatment response, assessed radiologically, was defined as complete response (CR), partial response (PR), stable disease (SD), and disease control rate (CR+PR+SD).^12–14^

Radiologic imaging, including computed tomography scans of the abdomen, pelvis, and chest, were performed every 3 months, and patients who underwent liver-directed therapy also received dedicated magnetic resonance imaging (MRI) along with an additional MRI scan within 8–12 weeks of the procedure. An octreotide scan was performed only at baseline and 6–12 months later to confirm disease progression and/or response. Serologic tumor markers were evaluated at each visit, and 5-HIAA was collected in 24-h urine samples every 3–6 months.

## Results

### Patient characteristics

A total of 16 patients (11 female, 5 male; age range, 43–81 years; mean age, 64.3 years) with nonfunctional and low-grade NETs were included in this retrospective chart review (Table 1). The primary tumor locations are presented in Table 1 and included 9 intestinal, 4 pancreatic, and 3 unknown. The locations of metastatic sites, along with stage (all of which are III or IV) and histology, are also presented in Table 1. Each of the 16 patients had been receiving octreotide LAR 30–60 mg for a median duration of 6.5 months (range 3–36 months) and were transitioned to lanreotide because of patient decision (*n* = 6), disease progression (*n* = 6), AEs (*n* = 2), poor tolerance (*n* = 1), and injection discomfort/pain (*n* = 1; Table 2). The starting doses of lanreotide were 120 mg (*n* = 13), 90 mg (*n* = 1), or 60 mg (*n* = 2) every 28 days, based on renal dysfunction. At the time the results reported here were quantified, the median number of lanreotide cycles was 5.23 (range 2–10); however, all but 1 patient is still receiving lanreotide. Concomitant therapies were noted in 8 of 16 patients, including radiofrequency ablation (*n* = 3), yttrium-90 transarterial radioembolization (Y^90^ TARE; *n* = 1), chemotherapy (*n* = 1), surgery (*n* = 1), chemotherapy+Y^90^ TARE (*n* = 1), and chemotherapy+surgery (*n* = 1; Table 3).

**Table 1.** **Baseline and Demographic Information (*N* = 16)**

Patient	Gender/age (years)	Primary tumor location	Metastatic site(s)	Grade	Stage (I–IV)	Histology^a^	Mitotic index (10 HPF)	Ki-67 (%)
1	M, 81	Duodenum	Liver, mesentery	G1	IV	Well-differentiated NET	<2	3
2	F, 68	Antrum of stomach	N/A	G1	III	Well-differentiated NET	<2	5
3	M, 69	Ileocecum	Right inguinal LN, liver	G2	IV	Moderately-differentiated, intermediate-grade NET	8–10	3–4
4	M, 81	Mesenteric mass (primary unknown)	Abdominal LN	G1	III	Well-differentiated NET, positive immunohistochemical stains for synaptophysin, chromogranin-A, serotonin, and negative for TTF-1	<2	7
5	F, 72	Ileum	LN, liver	G2	IV	Well- to moderately-differentiated NET	10	<10
6	F, 56	Unknown	Liver	G1	IV	Well-differentiated NET, positive immunohistochemical stains for synaptophysin, chromogranin-A, and negative for TTF-1, CK7, CK20, CDX2	6–8	8
7	F, 75	Mediastinal LN (primary unknown)	Hilar LN	G2	IV	Moderately-differentiated NET, positive immunohistochemical stains for synaptophysin, chromogranin-A, serotonin, and negative for TTF-1, CK7, VIP, S-100, CK20, CDX2, CK5/6, p63, napsin-A	14–15	26
8	M, 78	Pancreas	Liver, spleen	G2	IV	Moderately-differentiated NET	5–6	15
9	F, 60	Ileocecal	Liver	G2	IV	Moderately-differentiated NET	8	16
10	F, 53	Pancreas	Liver	G1	IV	ACTH-producing pancreatic NET complicated by Cushing's syndrome	8	N/A
11	F, 64	Gastric	Liver, spleen, bone, lung	G2	IV	Moderately-differentiated NET	4	10
12	F, 61	Pancreas	Peritoneum	G1	IV	Well-differentiated NET	1	N/A
13	M, 47	Ileum	Liver, colon	G1–G2	IV	Well- to moderately-differentiated NET	12	16
14	F, 43	Gastric	Liver	G2	IV	Moderately-differentiated NET	3	3
15	F, 56	Pancreas	Liver, LN	G1–G2	IV	Well- to moderately-differentiated NET	6	13
16	F, 64	Appendix	Peritoneum, LN	G2	IV	Moderately-differentiated NET	<2	4

^a^ Poorly, moderately, or well differentiated.

ACTH, adrenocorticotropic hormone; CDX2, caudal-type home box transcription factor 2; CK, cytokeratin; G, grade; HPF, high-power field; LN, lymph nodes; N/A, not assessed; NET, neuroendocrine tumor; TTF-1, thyroid transcription factor-1; VIP, vasoactive intestinal peptide.

**Table 2.** **Octreotide to Lanreotide Depot: Rationale and Dosing**

Patient	Last octreotide dose (mg IM)	Reason for transitioning to lanreotide	Lanreotide starting/current dose (mg SQ)	Total lanreotide doses
1	30	Patient decision	120	9
2	30	Patient decision	120	5
3	30	Patient decision	120	8
4	30	Patient decision	60^a^	8
5	30 to >40	Increased serologic marker+new liver lesion	120	8
6	30	Patient decision	120	3^b^
7	40	Diarrhea and abdominal pain	120	2 (stopped)^c^
8	30 to >40	Progressive disease (liver)	120	3
9^d^	20	Cost, GI pain, nausea	120	10
10	20	Radiologic and serologic disease progression	90^a^	5
11	30 to >60	GI upset, bone progression (stable liver)	120	3
12	40	Serologic marker	120	3
13	20 to >40	Patient decision	120	5
14	30	Intolerance, low muscle mass (anorexia)	120	3
15	Unknown	Serologic progression	120	3
16	30	Buttock pain	60^a^	6

^a^ Patients No. 4 and No. 10 were successfully escalated to full dose without any adverse events, and patient No. 16 had dose escalated to 90 mg but not beyond because kidney function remained moderately impaired due to diabetic nephropathy.

^b^ Past intolerance to octreotide-associated diarrhea.

^c^ Patient received 1 dose at Roswell Park Cancer Center (Buffalo, NY) and an unknown number of additional doses administrated by a healthcare professional located closer to the patient's residence.

^d^ Received 1 inadvertent misadministration of lanreotide depot by IM route instead of SQ route.

GI, gastrointestinal; IM, intramuscular, SQ, subcutaneous.

**Table 3.** **Biomarkers, Concurrent Treatments, Radiologic Response, and Serologic Markers**

Patient		CgA^a^ (nmol/L)	5-HIAA^b^ (μmol/day)	5-HT^c^ (nmol/L)	Required concurrent treatment?	Radiologic response	Other serologic markers
1	Level at lanreotide initiation	30	N/A	431.3	No	SD	Gastrin level reduced (<100 ng/L)^d^
	Current	39	N/A	448.3			
2	Level at lanreotide initiation	43	N/A	N/A	No	SD	Gastrin level reduced (239–76 ng/L)^d^
	Current	15	N/A	N/A			
3	Level at lanreotide initiation	7	N/A	2116.8	RFA	PR	None
	Current	<5	N/A	1509.6			
4	Level at lanreotide initiation	224	N/A	2451.6	No	SD	None
	Current	112	N/A	584.5			
5	Level at lanreotide initiation	18	25.1	3893.1	Y^90^ TARE	PR	None
	Current	14	18.8	3024.8			
6	Level at lanreotide initiation	366	29.8	505.1	No	SD	None
	Current	224	20.4	402.9			
7	Level at lanreotide initiation	168	N/A	<56.8	No	SD	None
	Current	122	N/A	<56.8			
8	Level at lanreotide initiation	2555	N/A	3961.2	CAPTEM and Y^90^ TARE	PR	None
	Current	204	N/A	505.1			
9	Level at lanreotide initiation	172	N/A	317.8	Debulking surgery	SD	None
	Current	29	N/A	431.3			
10	Level at lanreotide initiation	30	N/A	1163.4	No	SD	ACTH-producing tumor normalized; PPP reduced by 50% (1221–610 ng/L)^e^
	Current	<5	N/A	1140.7			
11	Level at lanreotide initiation	382	45.5	4523.0	CAPTEM, sunitinib	PD	None
	Current	160	16.2	1957.9			
12	Level at lanreotide initiation	67	N/A	1004.5	5-FU, irinotecan, debulking surgery	CR	PPP reduced by >50% (1100–430 ng/L)^e^
	Current	>5	N/A	862.6			
13	Level at lanreotide initiation	93	36.1	7320.8	RFA	PR	None
	Current	>5	21.4	1163.4			
14	Level at lanreotide initiation	21	17.3	3637.7	RFA	PR	None
	Current	18	66.9	164.6			
15	Level at lanreotide initiation	735	120.3	317.8	No	SD	None
	Current	440	83.7	187.3			
16	Level at lanreotide initiation	382	N/A	1339.3	No	SD	None
	Current	78	N/A	38.6			

^a^ CgA target level, 0–5.0 nmol/L.

^b^ 5-HIAA target level, 0–77.9 μmol/day.

^c^ 5-HT target level, 119.2–1821.7 nmol/L.

^d^ Gastrin normal range, 0–100 ng/L.

^e^ PPP normal range, 70–430 ng/L.

5-FU, 5-fluorouracil; 5-HIAA, 5-hydroxyindoleacetic acid; 5-HT, serotonin; CAPTEM, capecitabine+temozolomide; CgA, chromogranin A; CR, complete response; G1, grade 1; PD, progressive disease; PPP, pancreatic polypeptide; PR, partial response; RFA, radiofrequency ablation; SD, stable disease; TARE, transarterial radioembolization; Y^90^, yttrium-90.

### Adverse events

During the observation period, AEs affected 11 of the patients; gastrointestinal AEs (diarrhea, nausea, constipation, and abdominal pain) were the most common (*n* = 6) and were considered to be associated with lanreotide (Table 4). Other AEs experienced by more than 1 individual included fatigue (*n* = 3), hypertension (*n* = 2), hyperglycemia (*n* = 2), and pancreatic exocrine enzyme deficiency (*n* = 2).

**Table 4.** **Adverse Events**

Patient	Adverse events/grade (CTCAEv4.0)
1	G1 fatigue
2	None
3	G2 hypertension
4	None
5	G2 pancreatic exocrine enzyme deficiency, G1 weight loss^a^, G1 headache
6	None
7	G1 tremor, G2 hyperglycemia, G1 fatigue, G2 nausea, G1 blurred vision
8	G1 nausea, G1 abdominal pain
9	G1 diarrhea, G1 hypertension
10	None
11	G1 diarrhea
12	None
13	G1 diarrhea, G2 pancreatic exocrine enzyme deficiency, G3 hyperglycemia
14	G1 pruritus (no rash)
15	G1 fatigue
16	G1 constipation, G1 alopecia

^a^ Possibly related to pancreatic exocrine enzyme deficiency.

CTCAEv.4.0, Common Terminology Criteria for Adverse Events, version 4.0; G, grade 1.

### Tumor response

Radiologic treatment responses of the combination of lanreotide with other therapeutic modalities were CR (*n* = 1), PR (*n* = 5), or progressive disease (*n* = 1). Overall disease control (a combination of CR+PR+SD) was achieved in 15 of 16 patients (93.8%) at the end of the study. Five patients achieved PR; however, it is important to note that all of these patents also received concomitant liver-directed treatment: radiofrequency ablation (RFA) in 3 patients and Y^90^ TARE in 2 patients. One patient had no evidence of disease after surgical resection (CR). In addition to these patients, 9 others still had SD at the end of the study period.

### Biochemical response

Numerical decreases in CgA levels after initiation of lanreotide treatment were observed in 15 of 16 patients (range of CgA levels changed from 7–2555 nmol/L at baseline to <5–440 nmol/L after treatment; Table 3). Urinary 5-HIAA values, available for 7 patients, ranged from 17.3–120.3 μmol/day at baseline to 16.2–83.7 μmol/day after treatment. Serum serotonin ranged from <56.8–7320.8 nmol/L at baseline to <56.8–3024.8nmol/L following initiation of lanreotide. Decreases in other serologic markers were noted in 4 patients, including gastrin (*n* = 2), pancreatic polypeptide (*n* = 1), and pancreatic polypeptide and adrenocorticotropic hormone (*n* = 1).

## Discussion

This retrospective analysis reports the safety, tolerability, and efficacy of lanreotide in patients with various GEP-NETs who were previously treated with octreotide LAR. Lanreotide was well tolerated among these patients, including those who experienced disease progression or lack of tolerance on octreotide LAR. As a part of multidisciplinary management of these patents, many of them received concomitant treatment with other modalities, especially liver-directed therapy. Overall disease control (a combination of CR+PR+SD) was >90% in our study, including CR in 6%, PR in 31%, and SD in 56%. One patient had no evidence of disease after surgical resection (CR), while the patients with PR had received concurrent liver-directed therapy as described in the Results section. In 2016, Phan et al. reported on tumor response in the CLARINET study of lanreotide depot versus placebo in patients with metastatic GEP-NETs. Among 110 patients who were on the lanreotide arm, 2 patients achieved a PR and 65 demonstrated SD (44/103 patients receiving placebo).^15^

The most common AEs reported in these patients were consistent with those reported in previous studies of lanreotide.^4,5^ These results also noted the use of lanreotide in a patient with moderate renal dysfunction. In this patient, lanreotide was started at 60 mg and was titrated up to a maximum tolerated dose of 90 mg. This is noteworthy because dose adjustments of lanreotide depot for moderate renal impairment are currently only recommended for acromegaly patients.^11^

The outcomes of this study also demonstrated best tumor response of SD, PR, or CR in all but 1 patient during lanreotide treatment. Of note is that this case series included a small number of patients with GEP-NETs, which is a limitation, as is the retrospective nature of the analysis. Also, half of the patients (8/16) were receiving concomitant therapies, mostly liver-directed therapies, including radiofrequency ablation and transarterial radioembolization. Several patients received concomitant chemotherapy. Among these patients, gastrointestinal AEs, including diarrhea, were either absent or reported as low grade despite receiving concomitant chemotherapy containing capecitabine or irinotecan, 2 of the well-known systemic cytotoxic agents associated with dose-limiting toxicities, including diarrhea.

Following sequencing from octreotide to lanreotide, reductions in the levels of CgA were observed in 15 of 16 patients, with the most substantial reductions occurring among those who had the greatest levels before treatment with lanreotide. Currently, CgA, which can be identified in patients with GEP-NETs by immunohistochemistry, is widely used as a neuroendocrine marker, especially among patients with well-differentiated NETs.^16–18^ Despite its utility, the use of CgA has some limitations, including a lack of international standardization.^16^ In addition, recent research has raised a question of whether CgA acts more effectively as a diagnostic biomarker than as a method for identifying the risk of metastasis.^17^ However, the results of a pharmacokinetic/pharmacodynamics model analysis using CLARINET data suggest that change in CgA over time is a relevant covariate/predictor of PFS in GEP-NETs among patients who are either untreated or treatment-naive^18^; similar findings have been reported elsewhere.^12^

The efficacy of utilizing 5-HIAA as a prognostic marker among individuals with GEP-NETs has not been definitively established. Several studies have concluded that increased levels of urinary 5-HIAA can be associated with decreases in survival.^19,20^ However, a multivariate analysis published in 2016 indicated that there is no prognostic value for 5-HIAA.^21^ These confounding results clearly indicate that additional research regarding prognostic markers is still needed.

The prescribing information of lanreotide produced in 2014 includes renal dose adjustments for acromegaly,^11^ which should be considered when treating patients. Specifically, acromegaly patients with moderate to severe renal impairment should receive an initial dose of 60 mg by deep subcutaneous injection every 4 weeks for 3 months, followed by dose adjustments, as described for non–renal-impaired patients. It is stated that caution should be used when considering an extended dosing interval for acromegaly, including 120 mg every 6 or 8 weeks, among patients with moderate or severe renal impairment. However, no dose adjustments are recommended for mild to moderate renal impairment in patients with GEP-NETs, and currently there are no data available for dosing recommendations in GEP-NET patients with severe renal impairment.

Additional studies not only provide information pertaining to severe renal impairment but also help define efficacy and safety in patients who were previously treated with octreotide or another SSA. We are currently conducting a retrospective medical chart review that is designed to include data obtained from ∼100 patients (ClinicalTrials.gov number NCT03112694). The objectives of that retrospective analysis include evaluating the efficacy and safety of lanreotide in a real-world setting, providing additional information on lanreotide efficacy and safety following treatment with octreotide, assessing both quality of life and patient satisfaction, and identifying the reasons why patients transitioned from octreotide to lanreotide.

In summary, treatment with lanreotide was well tolerated among patients with GEP-NETs when given alone or in combination with other treatment modalities, including radioembolization, radiofrequency ablation, and chemotherapy. Dose escalation was also well tolerated in a patient with moderate renal dysfunction. To our knowledge, results from this retrospective analysis also documented for the first time a clinical, biochemical, and radiological benefit of transitioning patients with GEP-NETs from one SSA (octreotide LAR) to another (lanreotide).

## Abbreviation Used

5-HIAA: 5-hydroxyindoleacetic acid
AE: adverse event
CgA: chromogranin A
CI: confidence interval
CLARINET: Controlled Study of Lanreotide Antiproliferative Response in Neuroendocrine Tumors
CR: complete response
FDA: Food and Drug Administration
GEP-NETs: gastroenteropancreatic neuroendocrine tumors
LAR: long-acting release
MRI: magnetic resonance imaging
NETs: neuroendocrine tumors
PFS: progression-free survival
PR: partial response
SD: stable disease
SSAs: somatostatin analogs
Y^90^ TARE: yttrium-90 tranarterial radioembolization

## References

[1] Dasari A, Shen C, Halperin D, et al. Trends in the incidence, prevalence, and survival outcomes in patients with neuroendocrine tumors in the United States. JAMA Oncol. 2017;3:1335–1342 28448665 10.1001/jamaoncol.2017.0589 PMC5824320

[2] Reubi JC, Schonbrunn A Illuminating somatostatin analog action at neuroendocrine tumor receptors. Trends Pharmacol Sci. 2013;34:676–688 24183675 10.1016/j.tips.2013.10.001 PMC3883302

[3] Qian ZR, Li T, Ter-Minassian M, et al. Association between somatostatin receptor expression and clinical outcomes in neuroendocrine tumors. Pancreas. 2016;45:1386–1393 27622342 10.1097/MPA.0000000000000700 PMC5067972

[4] Caplin ME, Pavel M, Ćwikła JB, et al. Lanreotide in metastatic enteropancreatic neuroendocrine tumors. N Engl J Med. 2014;371:224–233 25014687 10.1056/NEJMoa1316158

[5] Caplin ME, Pavel M, Ćwikła JB, et al. Anti-tumour effects of lanreotide for pancreatic and intestinal neuroendocrine tumours: The CLARINET open-label extension study. Endocr Relat Cancer. 2016;23:191–199 26743120 10.1530/ERC-15-0490 PMC4740728

[6] Vinik AI, Wolin EM, Liyanage N, et al. Evaluation of lanreotide depot/autogel efficacy and safety as a carcinoid syndrome treatment (ELECT): A randomized, double-blind, placebo-controlled trial. Endocr Pract. 2016;22:1068–1080 27214300 10.4158/EP151172.OR

[7] Cives M, Strosberg J The expanding role of somatostatin analogs in gastroenteropancreatic and lung neuroendocrine tumors. Drugs. 2015;75:847–858 25911185 10.1007/s40265-015-0397-7

[8] Saif MW Lanreotide for the treatment of gastroenteropancreatic neuroendocrine tumors. Expert Opin Pharmacother. 2016;17:443–456 26635177 10.1517/14656566.2016.1127914

[9] Sandostatin LAR^®^ Depot (Octreotide Acetate for Injectable Suspension). Novartis Pharmaceuticals Corp.: East Hanover, NJ, 2014

[10] Rinke A, Müller HH, Schade-Brittinger C, et al. Placebo-controlled, double-blind, prospective, randomized study on the effect of octreotide LAR in the control of tumor growth in patients with metastatic neuroendocrine midgut tumors: A report from the PROMID Study Group. J Clin Oncol. 2009;27:4656–4663 19704057 10.1200/JCO.2009.22.8510

[11] Somatuline^®^ Depot (lanreotide) Injection [Prescribing Information]. Ipsen Biopharmaceuticals, Inc.: Basking Ridge, NJ; 2017

[12] Bajetta E, Procopio G, Catena L, et al. Lanreotide autogel every 6 weeks compared with Lanreotide microparticles every 3 weeks in patients with well differentiated neuroendocrine tumors: A Phase III Study. Cancer. 2006;107:2474–2481 17054107 10.1002/cncr.22272

[13] Bajetta E, Zilembo N, Di Bartolomeo M, et al. Treatment of metastatic carcinoids and other neuroendocrine tumors with recombinant interferon-alpha-2a. A study by the Italian Trials in Medical Oncology Group. Cancer. 1993;72:3099–3105 7693327 10.1002/1097-0142(19931115)72:10<3099::aid-cncr2820721035>3.0.co;2-4

[14] Eisenhauer EA, Therasse P, Bogaerts J, et al. New response evaluation criteria in solid tumours: Revised RECIST guideline (version 1.1). Eur J Cancer. 2009;45:228–247 19097774 10.1016/j.ejca.2008.10.026

[15] Korse CM, Taal BG, Vincent A, et al. Choice of tumour markers in patients with neuroendocrine tumours is dependent on the histological grade. A marker study of Chromogranin A, Neuron specific enolase, Progastrin-releasing peptide and cytokeratin fragments. Eur J Cancer. 2012;48:662–671 21945100 10.1016/j.ejca.2011.08.012

[16] Phan AT, Dasari A, Liyanage N, et al. Tumor response in the CLARINET study of lanreotide depot vs. placebo in patients with metastatic gastroenteropancreatic neuroendocrine tumors (GEP-NETs). J Clin Oncol. 2016;34:434–434

[17] Tang C, Gong L, Zou W, et al. Multivariate analysis of metastasisrelated risk factors for patients with gastroenteropancreatic neuroendocrine tumors based on clinicopathological and endoscopic features. Oncol Rep. 2016;36:3343–3352 27748940 10.3892/or.2016.5170

[18] Buil-Bruna N, Dehez M, Manon A, et al. Establishing the quantitative relationship between lanreotide autogel^®^, chromogranin A, and progression-free survival in patients with nonfunctioning gastroenteropancreatic neuroendocrine tumors. AAPS J. 2016;18:703–712 10.1208/s12248-016-9884-3 PMC5256603 26908127

[19] Formica V, Wotherspoon A, Cunningham D, et al. The prognostic role of WHO classification, urinary 5-hydroxyindoleacetic acid and liver function tests in metastatic neuroendocrine carcinomas of the gastroenteropancreatic tract. Br J Cancer. 2007;96:1178–1182 17406366 10.1038/sj.bjc.6603699 PMC2360161

[20] van der Horst-Schrivers AN, Post WJ, Kema IP, et al. Persistent low urinary excretion of 5-HIAA is a marker for favourable survival during follow-up in patients with disseminated midgut carcinoid tumours. Eur J Cancer. 2007;43:2651–2657 17825550 10.1016/j.ejca.2007.07.025

[21] Zandee WT, Kamp K, van Adrichem RC, et al. Limited value for urinary 5-HIAA excretion as prognostic marker in gastrointestinal neuroendocrine tumours. Eur J Endocrinol. 2016;175:361–366 27491374 10.1530/EJE-16-0392

